# Integrative bioinformatics analysis and experimental validation reveals key genes and regulatory mechanisms in the development of gout

**DOI:** 10.3389/fgene.2025.1598835

**Published:** 2025-06-18

**Authors:** Ye Yuan, Zhiqiang Gao, Jianhong Chen, Yuejing Liu, Jingguo Zhou

**Affiliations:** ^1^ Department of General Practice, Hospital of Chengdu University of Traditional Chinese Medicine, Chengdu, China; ^2^ Clinical Medical College, Chengdu University of Traditional Chinese Medicine, Chengdu, China; ^3^ Geriatrics Department, Hospital of Chengdu University of Traditional Chinese Medicine, Chengdu, China; ^4^ Department of Rheumatology and Immunology, The First Affiliated Hospital of Chengdu Medical College, Chengdu, China.

**Keywords:** gout, ceRNA network, immune infiltration, molecular docking, apoptosis, inflammatory

## Abstract

**Background and Aims:**

Gout is a prevalent inflammatory arthropathy caused by monosodium urate crystal deposition, yet its molecular pathogenesis remains incompletely understood. This study aimed to identify key genes and elucidate regulatory mechanisms underlying gout development through bioinformatics analysis combined with experimental validation.

**Methods:**

Transcriptome dataset GSE160170 and single-cell dataset GSE211783 were analyzed using differential expression analysis and weighted gene co-expression network analysis (WGCNA). Functional enrichment, protein-protein interaction (PPI), ceRNA, and transcription factor networks were constructed. Immune cell infiltration was analyzed using CIBERSORTx. Molecular docking predicted therapeutic compounds. Experimental validation included qRT-PCR, Western blot, gene knockdown/overexpression, and functional assays.

**Results:**

Among 329 gout-related genes identified, CXCL8, PTGS2, and IL10 emerged as key regulators involved in cell-cell adhesion, leukocyte activation, and NF-κB signaling. Immune infiltration revealed significant upregulation of M2 macrophages, activated mast cells, activated NK cells, and γδ T cells in gout samples. CeRNA network identified KCNQ1OT1 and hsa-mir-98-5p as regulatory elements, while CEBPB, STAT3, RELA, and NFKB1 were key transcription factors. Molecular docking suggested pergolide as a therapeutic candidate. Single-cell analysis confirmed high expression of key genes in T/NK cells and myeloid cells. Western blot validation showed upregulated protein expression of key genes in the gout model. PTGS2 knockdown enhanced cell viability and reduced apoptosis, while overexpression promoted inflammatory cytokine production and NF-κB pathway activation.

**Conclusion:**

This study systematically elucidated the pivotal roles of CXCL8, PTGS2, and IL10 in gout pathogenesis, providing valuable molecular targets for therapeutic development.

## 1 Introduction

Gout is a prevalent and complex form of inflammatory arthritis that results from the deposition of monosodium urate (MSU) crystals in joints and surrounding tissues (Gu et al., 2023). The formation of MSU crystals occurs when serum uric acid levels exceed the physiological saturation threshold, leading to the precipitation of urate crystals (Gu et al., 2023). The deposition of these crystals triggers a cascade of inflammatory responses, including the activation of resident macrophages, recruitment of neutrophils, and production of pro-inflammatory cytokines such as interleukin-1β (IL-1β), tumor necrosis factor-α (TNF-α), and interleukin-6 (IL-6) (Gu et al., 2023; Galozzi et al., 2021). Clinically, this acute inflammation presents as gout flares, marked by severe pain, swelling, and joint erythema. Recurrent acute flares can progress to chronic tophaceous gout, characterized by tophi formation in joints, tendons, and soft tissues, leading to persistent inflammation, joint damage, and deformity, greatly affecting patients’ quality of life (Tao et al., 2023). Furthermore, hyperuricemia and MSU crystal deposition in the kidneys can cause uric acid nephrolithiasis and chronic kidney disease (Kim et al., 2016). The global burden of gout has been increasing in recent decades, with an estimated prevalence of 1%–4% in developed countries (Singh and Gaffo, 2020). This rising prevalence has been attributed to various factors, including aging populations, increased prevalence of comorbidities such as obesity and metabolic syndrome, and changes in dietary habits (Singh and Gaffo, 2020; Pai et al., 2024). Gout is more common in men than women and tends to occur more frequently in older age groups (Dehlin et al., 2020). Despite significant progress in elucidating the pathophysiology of gout, the precise molecular mechanisms underlying the disease remain incompletely understood.

In recent years, high-throughput sequencing technologies and bioinformatics analyses have provided powerful tools for unraveling the complex molecular networks underlying human diseases (Uesaka et al., 2022). Transcriptome profiling, which measures genome-wide gene expression levels, has been widely used to identify differentially expressed genes (DEGs) and dysregulated pathways in various diseases, including gout. Weighted gene co-expression network analysis (WGCNA) is a systems biology approach that constructs gene co-expression networks to identify key gene modules and hub genes associated with clinical traits. Integration of differential expression analysis and WGCNA can robustly identify disease-related genes and pathways. Moreover, the role of competing endogenous RNAs (ceRNAs) and transcription factors (TFs) in the regulation of key genes in gout remains largely unknown. The ceRNA hypothesis proposes that long non-coding RNAs (lncRNAs) can act as microRNA (miRNA) sponges to regulate the expression of mRNAs, forming a complex regulatory network (Wang et al., 2021). TFs are also important regulators of gene expression that can activate or repress transcription by binding to specific DNA sequences (Wagh et al., 2023). Investigating the ceRNA and TF regulatory networks in gout may provide novel insights into the molecular mechanisms of this disease. In addition, recent advances in single-cell RNA sequencing (scRNA-seq) technology have enabled the characterization of the transcriptome at the individual cell level, providing unprecedented resolution for understanding the cellular heterogeneity and dynamics in complex tissues (Conte et al., 2024). scRNA-seq has been applied to study various rheumatic diseases, such as rheumatoid arthritis (Su et al., 2024) and systemic lupus erythematosus (Hu et al., 2024), revealing novel cell subpopulations and their roles in disease pathogenesis. However, the application of scRNA-seq in gout research is still limited.

Therefore, in this study, we aimed to systematically investigate the key genes, pathways, and regulatory mechanisms underlying the pathogenesis of gout by integrating bulk RNA-seq and scRNA-seq data using bioinformatics methods. We identified differentially expressed genes between gout patients and healthy controls and constructed a weighted gene co-expression network to identify gout related genes. Functional enrichment analysis revealed the biological processes and pathways enriched in the key genes. We further constructed a protein-protein interaction network to identify key genes associated with gout and validated their diagnostic performance. A nomogram risk prediction model was built based on the key genes. Moreover, we analyzed the immune cell infiltration landscape in gout and its correlation with key genes. A ceRNA network was constructed to explore the regulatory interactions among lncRNAs, miRNAs, and key genes. Transcription factors regulating the key genes were also predicted. CMap analysis and molecular docking were performed to identify potential small molecule drugs targeting the key genes. Finally, we utilized single-cell transcriptome data to validate the expression patterns of key genes in different cell types.

## 2 Materials and methods

### 2.1 Data acquisition and preprocessing

Gout-related datasets (GSE160170 and GSE211783) were downloaded from the Gene Expression Omnibus (GEO) database (https://www.ncbi.nlm.nih.gov/geoprofiles/). Among them, the GSE160170 dataset is based on GPL21827 (Agilent-079487 Arraystar Human LncRNA microarray V4), including transcriptome data of peripheral blood samples from 6 gout patients and 6 normal individuals. GSE211783 is a single-cell transcriptome dataset based on the GPL24676 platform (Illumina NovaSeq 6000), including peripheral blood samples from 3 gout patients and 3 normal individuals.

These datasets were preprocessed. For the transcriptome dataset GSE160170, the R software “affy” package was used for standardized processing, and log2 (matrix +1) was used for standardization. For the single-cell transcriptome dataset GSE211783, the 10x Genomics Cell Ranger software was used for alignment, quantification and cell identification, and then the Seurat package was used to read in the gene-cell expression matrix, and high-quality cells were screened for subsequent analysis based on the number of genes detected in each cell (nFeature_RNA) between 500 and 7000. Specifically, the gene expression of each cell was normalized by the “LogNormalize” method, and highly variable genes were selected as feature genes for subsequent analysis. Secondly, the expression variance of each gene was calculated by the “FindVariableFeatures” function, and the top 2000 genes with the highest variance were selected as feature genes for downstream clustering analysis. Then, the expression matrix of feature genes was scaled and centralized using the “ScaleData” function, and PCA (Principal Component Analysis) was used for dimensionality reduction.

### 2.2 Preselection of diagnostic biomarker

#### 2.2.1 Differential expression analysis

The R software package “limma” was used for transcriptome data, with the threshold of “fold change of 2 and P < 0.05,” to perform differential gene expression analysis on gout group samples and normal control group samples in the training set GSE160170, and screen out differentially expressed genes (DEGs).

#### 2.2.2 Weighted gene co-expression network analysis (WGCNA)

The R software package “WGCNA” was used to perform weighted gene co-expression network analysis (WGCNA) on the transcriptome data. First, the pickSoftThreshold function was used to analyze the scale-free fitness index and average connectivity to determine the soft threshold power β. The optimal soft threshold was selected to construct the adjacency matrix, which was then converted into a topological overlap matrix (TOM) to measure the co-expression similarity between genes. Then, hierarchical clustering and dynamic tree cutting algorithm were used to divide TOM, and multiple gene modules were identified. Then, the correlation between the first principal component of each module - module eigengenes (MEs) and the clinical grouping (NC group or Cout group) was analyzed to identify gene modules significantly related to disease occurrence and development (*P* < 0.05). Finally, the gene significance (GS) of each module was calculated. With the threshold of “GS > 0.5 and *P* < 0.05,”, hub genes were screened from the co-expression modules significantly related to the occurrence and development of gastric cancer. By comparing the differentially expressed genes and the hub genes under WGCNA, common genes were screened, and these genes were considered to be closely related to the occurrence and development of gout.

### 2.3 Functional enrichment analysis

GO and KEGG enrichment analysis was performed on the common genes using the clusterProfiler package. GO was used to annotate the biological processes, molecular functions and cellular components of genes. KEGG was used to annotate gene pathways. Enrichment was considered statistically significant when P < 0.05. Based on the Gene Ontology (GO) (http://geneontology.org/) and Kyoto Encyclopedia of Genes and Genomes (KEGG) (http://www.kegg.jp/) databases, functional enrichment analysis was performed. Among them, GO functional enrichment analysis was performed from three dimensions: biological process (BP), cellular component (CC) and molecular function (MF). To further explore abnormally expressed signaling pathways in pathological conditions and verify the accuracy of functional enrichment analysis, based on the transcriptome data GSE160170, we used the R software “gsea” package to analyze Gene Set Enrichment Analysis (GSEA) to further compare the expression differences of signaling pathways in the enrichment analysis between the Cout and NC groups, so as to verify the accuracy of functional enrichment analysis. The normalized enrichment score (NES) and P value were used to evaluate the expression changes of related pathways, with P < 0.05 indicating significant expression differences in signaling pathways.

### 2.4 PPI network analysis

The candidate common genes were input into the String platform (https://string-db.org/), and independent genes were removed. Cytoscape was used to screen key genes and establish a protein-protein interaction (PPI) network. The above genes were used for Cytoscape software, and the MCC algorithm (identification of central objects and subnetworks from complex interaction sets) was used to calculate the TOP10 genes in the PPI network. The TOP10 genes were screened using the DMCN algorithm, and the intersection of the two algorithms was taken to screen common genes, which were considered to be key genes closely related to the occurrence and development of gout.

### 2.5 Expression and ROC analysis of key genes

The Wilcoxon rank-sum test was used to analyze the expression differences of key genes in gout group samples and normal control samples; then the R software “pROC” package was used for receiver operating characteristic (ROC) curve analysis to evaluate the diagnostic accuracy of each key gene for the disease. The area under the curve (AUC) of each gene was calculated, with values close to 1 indicating better diagnostic performance. In the methodological validation, we employed a combined approach of Bootstrap and Leave-One-Out Cross-Validation (LOOCV). Specifically, the original samples were resampled with replacement using the Bootstrap method to generate multiple training sets, and LOOCV was applied within each training set for model evaluation. By repeating the Bootstrap-LOOCV process multiple times and integrating the evaluation results, we were able to more robustly estimate the model’s performance and its distribution, thereby improving the reliability and generalizability of the results. This approach effectively reduces the randomness caused by sample partitioning and provides a solid data foundation for subsequent analyses.

### 2.6 Construction of nomogram risk prediction model

Based on the results of multivariate logistic regression analysis, we used the “rms” package of R software (version 4.1.0) to construct a nomogram prediction model using the screened significantly related key genes as predictors, and used the Bootstrap method (repeated sampling 1000 times) for internal validation to ensure model stability. Calibration curves were used to evaluate the performance of the model by comparing the predicted probability with the observed probability. The unreliability test was used to assess the consistency between the predicted probability and the observed probability. In addition, decision curve analysis (DCA) was also performed to evaluate the clinical utility of the nomogram by quantifying the net benefit at different threshold probabilities.

### 2.7 Immune infiltration analysis

CIBERSORTx is a machine learning algorithm based on gene expression characteristics that can infer the relative abundance of 22 human immune cell subsets in complex tissues. Here, we used the CIBERSORTx (https://cibersortx.stanford.edu/) database to analyze the type and relative proportion of immune cells in each gout sample and normal control sample. The Wilcoxon rank-sum test was used to compare the differences in immune cell infiltration between gout samples and normal controls. The Pearson test was used to detect the correlation between immune cells as well as between key genes and immune cells.

### 2.8 Construction of ceRNA network and prediction of transcription factors (TF)

To elucidate competitive endogenous RNA (ceRNA) regulatory interactions, a lncRNA-miRNA-mRNA network was constructed using the miRNet database (https://www.mirnet.ca/). Key genes were inputted into miRNet to predict upstream miRNAs, which were then used to identify upstream lncRNAs with competitive binding relationships. The comprehensive ceRNA network was visualized using Cytoscape (https://cytoscape.org/). Additionally, the TRRUST database (https://www.grnpedia.org/trrust/) was used to predict transcription factors regulating key gene expression, and a TF-mRNA regulatory network was constructed and visualized with Cytoscape software.

### 2.9 Drug prediction and molecular docking

The Connectivity Map (CMap) database (https://clue.io) was used to predict potential small molecules that may reverse gout-related gene expression patterns. Upregulated and downregulated genes were uploaded to the CMap platform for gene set enrichment analysis, with small molecules showing negative enrichment scores indicating potential therapeutic effects. For molecular docking validation, three-dimensional structure files of candidate drugs were obtained from the PubChem database (https://pubchem.ncbi.nlm.nih.gov/), and crystal structures of key gene-encoded proteins were downloaded from the RCSB PDB database (https://www.rcsb.org/). AutoDock Vina software (version 1.2.2) was used to calculate binding free energy and analyze binding modes between small molecules and target proteins. The ligand-receptor complexes with optimal binding interactions were selected for visualization to elucidate the interaction mechanisms.

### 2.10 Single-cell transcriptome analysis

After obtaining the dimensionality-reduced single-cell data, this study used the “FindNeighbors” and “FindClusters” functions to perform clustering analysis on the cells, and divided the cells into different subgroups through the shared nearest neighbor (SNN) graph and Louvain algorithm. To annotate each subgroup, this study used the “SingleR” package to compare with known cell type marker genes to identify the cell type of each cell subgroup. In addition, the “RunUMAP” function was used for nonlinear dimensionality reduction and visualization of cells, generating a Uniform Manifold Approximation and Projection (UMAP) plot to intuitively display the distribution and relationship of each cell subgroup. After identifying each cell subgroup, this study further analyzed the distribution differences of each cell type between the Gout group and the NC group, and identified the characteristic cells closely related to the occurrence and development of gout. In addition, this study analyzed the expression patterns of key genes in the Gout and NC groups by plotting UMAP plots, and analyzed the expression of key genes in each cell subgroup by plotting violin plots. Finally, the Wilcoxon rank-sum test was used to compare the expression differences of key genes between the Gout and NC groups.

### 2.11 Experimental verification for function of key genes

#### 2.11.1 Cell culture and model construction

Human joint synovial cells were purchased from the cell bank of the Chinese Academy of Sciences. The cells were cultured in DMEM medium containing FBS (20%, Gibco, Pleasantville, NY) and penicillin/streptomycin (1%, Solarbio, Beijing, China), at 37°C and 5% CO_2_ in an incubator. Additionally, MSU crystals (200 μg/mL) were used to treat the cells for 4–48 h to construct a gout model.

#### 2.11.2 qRT-PCR

Total RNA was extracted using TRIzol reagent (Invitrogen, United States) and reverse transcribed using PrimeScript RT reagent kit (Takara, Japan) following manufacturer’s protocol. qRT-PCR was performed using TB Green Premix Ex Taq with cycling conditions: 95°C for 30 s, followed by 40 cycles of 95°C for 5 s and 60°C for 30 s. Primer sequences were: GAPDH forward 5′-TGC​ACC​ACC​AAC​TGC​TTA​GC-3′, reverse 5′-ACT​GTG​GTC​ATG​AGT​CCT​TCC​A-3'; PTGS2 forward 5′-GCA​AAT​TGC​TGG​CAG​GGT​TG-3′, reverse 5′-GCT​CTG​GTC​AAT​GGA​AGC​CT-3′. Relative gene expression was calculated using 2^−ΔΔCt^ method with GAPDH as internal control.

#### 2.11.3 Western blot

Total protein was extracted from synovial cells using RIPA lysis buffer containing protease and phosphatase inhibitors. Protein concentrations were determined by BCA assay (Beyotime, Shanghai, China). Equal amounts of protein (30 μg) were separated by 10% SDS-PAGE and transferred to PVDF membranes (Millipore, Bedford, MA, United States). Membranes were blocked with 5% non-fat milk in TBST for 1 h at room temperature, then incubated overnight at 4°C with primary antibodies against PTGS2, IL10, p-NF-κB p65, NF-κB p65, and GAPDH (all from Proteintech, Shanghai, China). After washing, membranes were incubated with HRP-conjugated secondary antibodies for 1 h. Protein bands were visualized using ECL reagent and detected with the Tanon 5200 chemiluminescent imaging system (Tanon Science & Technology, Shanghai, China). Band intensities were quantified using ImageJ software with GAPDH as the loading control.

#### 2.11.4 Cell transfection

PTGS2 shRNA and overexpression vectors were synthesized by Sangon Biotech. Human synovial cells were transfected using Lipofectamine 3000 (Invitrogen, United States) at 70%–80% confluence according to manufacturer’s instructions. Cells were collected 48 h post-transfection. Groups included untransfected control, transfection control, sh-PTGS2, and OE-PTGS2.

#### 2.11.5 Enzyme-Linked immunosorbent assay (ELISA)

Cell culture supernatants were collected and inflammatory factors (IL-1β, TNF-α, IL-6) were detected using human ELISA kits (R&D Systems, United States). Absorbance at 450 nm was measured and concentrations calculated according to standard curves.

#### 2.11.6 Cell viability detection

Cell viability was assessed using CCK-8 kit (Dojindo, Japan). Cells were seeded in 96-well plates, treated for 24 h, then incubated with CCK-8 solution for 2 h at 37°C. Absorbance at 450 nm was measured to calculate viability percentages.

#### 2.11.7 Flow cytometry detection of cell apoptosis

Cell apoptosis was detected using Annexin V-FITC/PI double staining method. Cells from each group were collected, washed with PBS, and resuspended in Annexin V binding buffer. Annexin V-FITC and PI staining solutions were added, and after incubation at room temperature in the dark for 15 min, detection was performed on a flow cytometer (BD FACSCanto II, United States). Data were analyzed using FlowJo software to calculate the percentages of early and late apoptotic cells.

### 2.12 Statistical analysis

All experiments were repeated 3 times independently. Data were analyzed using SPSS 25.0 software and expressed as mean ± standard deviation (x̄±s). Two-group comparisons used t-test, and multiple-group comparisons used one-way ANOVA with Tukey’s *post hoc* test. *P* < 0.05 was considered statistically significant.

## 3 Results

### 3.1 Screening of differentially expressed genes in transcriptome data

**FIGURE 1:**
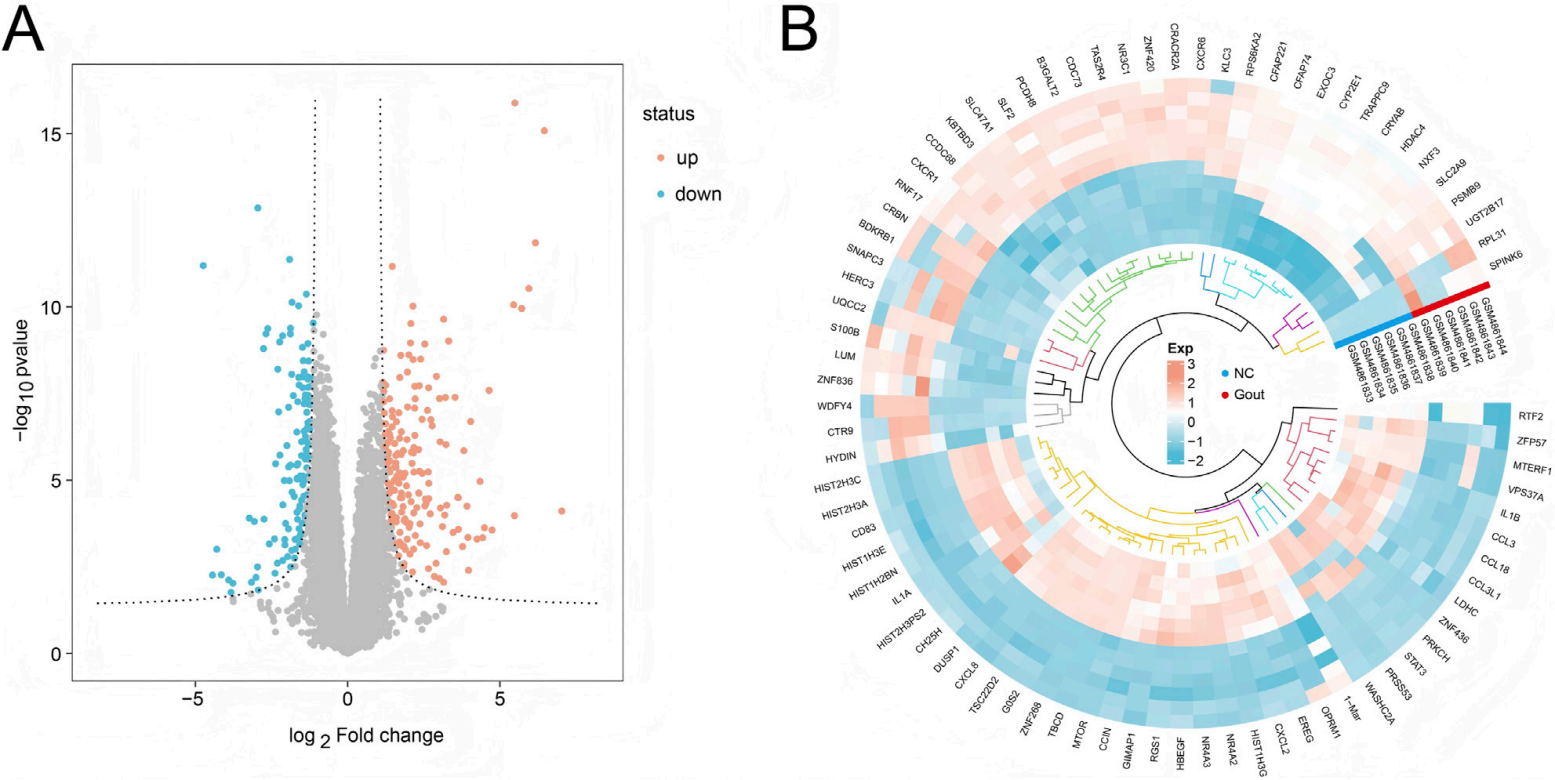
Differential expression analysis. **(A)** Volcano plot showing differentially expressed genes (DEGs) between the Gout group and the NC group. **(B)** Heatmap displaying the top 20 most significantly upregulated and downregulated genes in the Gout group compared to the NC group.

First, mRNA screening was performed on the GSE160170 dataset, and mRNA expression profile data related to gout disease were finally obtained. Subsequent data cleaning and normalization were performed on the expression profile data using the “limma” package for differential analysis. A total of 906 differentially expressed genes were screened, among which 342 genes were significantly upregulated in gout disease group samples (*P* < 0.05), and 564 genes were significantly downregulated in gout disease group samples (*P* < 0.05). The volcano plot of differential expression analysis is shown in Figure 1A, and the heat map of differentially expressed genes is shown in Figure 1B.

### 3.2 WGCNA

**FIGURE 2:**
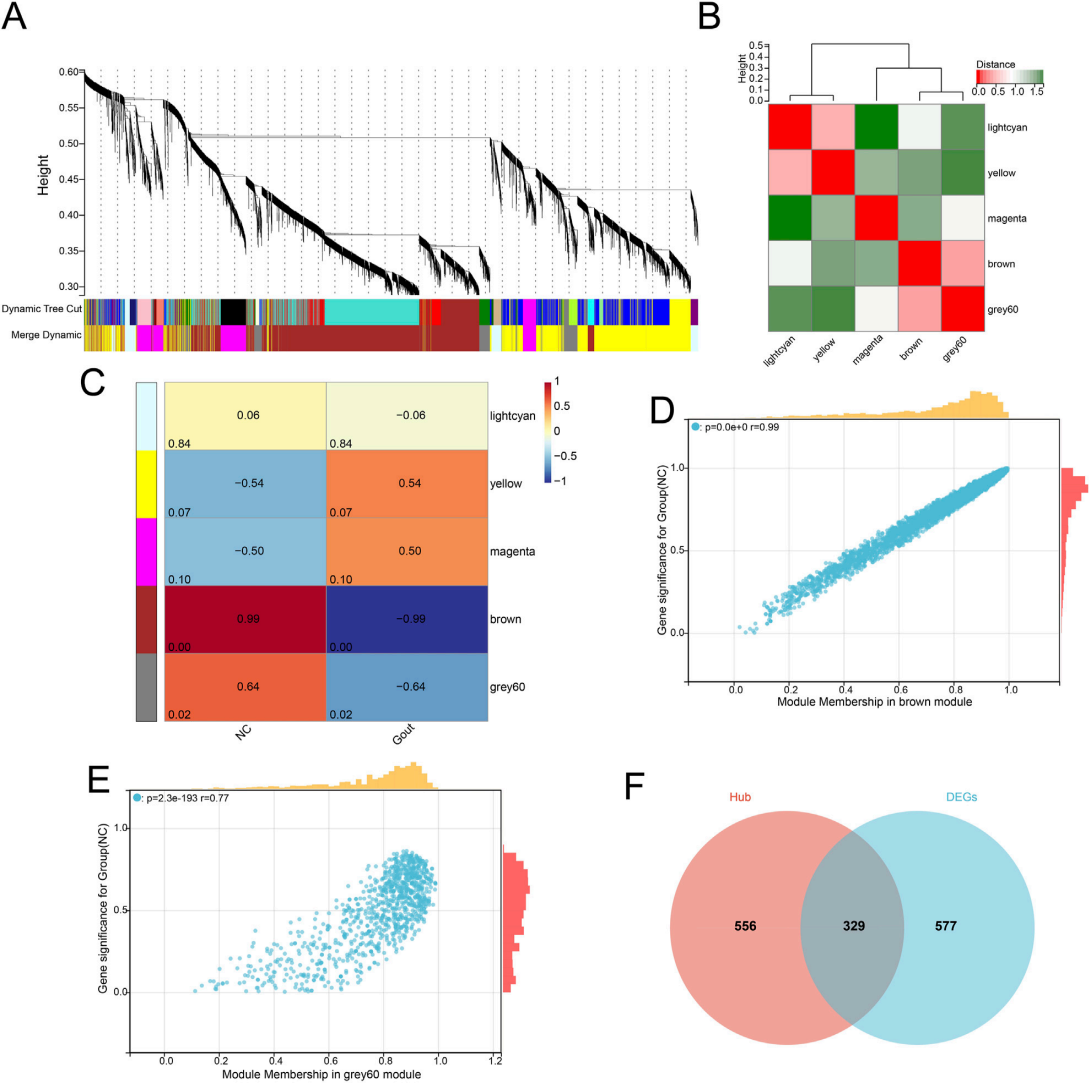
Weighted gene co-expression network analysis (WGCNA). **(A)** Cluster dendrogram of genes based on topological overlap. **(B)** Heatmap showing the correlation between module eigengenes. **(C)** Heatmap illustrating the correlation between modules and clinical traits (disease status). **(D)** Scatter plot of gene significance (GS) vs. module membership (MM) in the brown module. **(E)** Scatter plot of GS vs. MM in the grey60 module. **(F)** Venn diagram showing the overlap between hub genes identified by WGCNA and DEGs.

The pickSoftThreshold function was used to test different soft thresholds β, and an adjacency matrix was constructed based on the selected optimal soft threshold (β = 8). Then, based on hierarchical clustering and dynamic tree cutting algorithm, the minimum module gene number was set to 30, the deep split was set to 3, and the maximum module distance was set to 0.25, generating a total of 5 gene modules (Figure 2A). Then, the connectivity of MEs was analyzed, and the results showed that the distance between modules was greater than 0.25, indicating good independence between each module (Figure 2B). Then, the correlation between the feature genes of MEs and the disease grouping and trend was calculated. The results showed that a total of 2 gene modules were significantly correlated with the disease grouping regulation trend, namely, the Brown and grey60 modules, and 885 hub genes were identified from these gene modules (Figures 2C–E). By comparing the differentially expressed genes and the hub genes under WGCNA, the two were intersected, and a total of 329 common genes were identified (Figure 2F) which were considered to be genes closely related to the occurrence and development of gout.

### 3.3 Functional enrichment analysis

**FIGURE 3:**
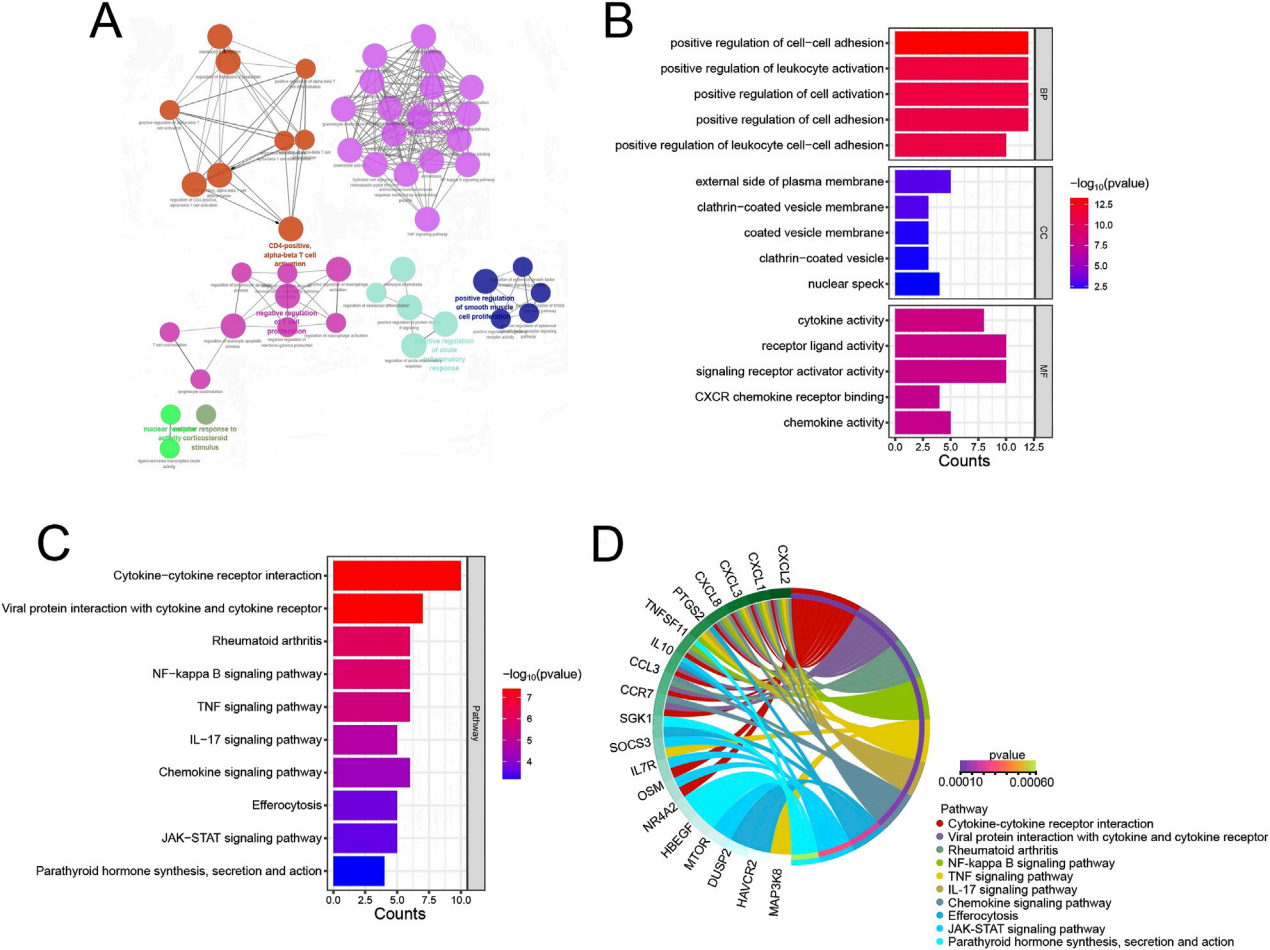
Functional enrichment analysis. **(A)** Network displaying the relationships among enriched GO terms and KEGG pathways. Each node represents a GO term or KEGG pathway, and edges represent the interactions between them. The node size is proportional to the number of genes in each term or pathway. **(B)** Bar plot showing the top 5 significantly enriched GO terms in the BP, CC, and MF categories. **(C)** Bar plot displaying the top 10 significantly enriched KEGG pathways. **(D)** Interaction relationship between KEGG signaling pathways and related genes.

GO an KEGG functional enrichment analysis was performed on the 35 marker genes screened from the above Module 1. First, GO and KEGG enrichment analysis was performed using the ClueGO plugin in Cytoscape (Figures 3A–D). Analysis of the GO enrichment process revealed that in BP, these genes were mainly enriched in positive regulation of cell-cell adhesion, positive regulation of leukocyte activation, positive regulation of cell activation, positive regulation of cell adhesion, positive regulation of leukocyte cell-cell adhesion, etc.; in CC, these genes were mainly enriched in external side of plasma membrane, clathrin-coated vesicle membrane, coated vesicle membrane, clathrin-coated vesicle, nuclear speck, etc.; in MF, these genes were mainly enriched in cytokine activity, receptor ligand activity, signaling receptor activator activity, CXCR chemokine receptor binding, chemokine activity, etc. (Figure 3B). KEGG enrichment analysis showed that these genes were involved in Ribosome, Glucagon signaling pathway, Carbon metabolism, African trypanosomiasis, Various types of N-glycan biosynthesis, Glycine, serine and threonine metabolism, Pyruvate metabolism, N-Glycan biosynthesis and other signaling pathways (Figure 3C).

### 3.4 PPI network interaction analysis

**FIGURE 4:**
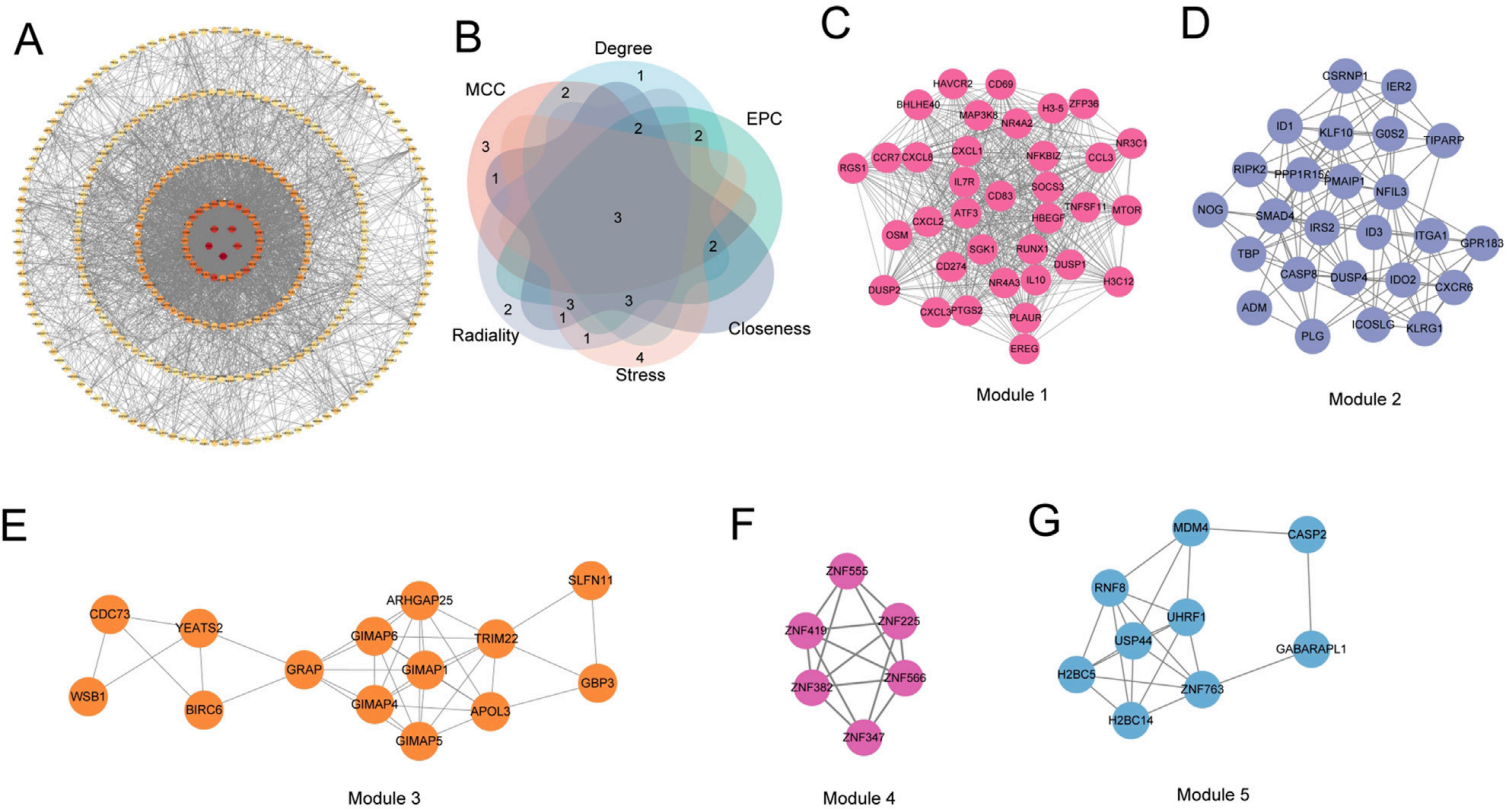
Protein-protein interaction (PPI) network analysis. **(A)** PPI network of common genes. **(B)** Venn diagram showing the overlap of marker genes identified by different algorithms (MCC, Degree, EPC, Closeness, Stress, and Radiality). **(C–G)** Subnetworks of gene clusters identified by the MCODE algorithm. Each subnetwork represents a densely connected module in the PPI network, with the MCODE score indicating the importance of the module.

Subsequently, the interaction relationship between the 329 encoded proteins was analyzed through the String database, and a PPI network was constructed (Figure 4A). Eleven independent genes were deleted from the network, and the remaining 318 genes were used for subsequent analysis. The average node degree (Dgree) of the network was 14.86, and the local clustering coefficient (Clustering Coefficient) of the network was 0.40, indicating a good interaction relationship between these candidate genes. Subsequently, the TOP15 marker genes selected by the MCC, Degree, EPC, Closeness, Stress and Radiality algorithms built into cytoscope were intersected, and 3 key genes (CXCL8, PTGS2 and IL10) were finally identified (Figure 4B). In addition, MCODE found that the PPI network of 318 common genes was mainly divided into five gene clusters, and the MCODE score of each gene cluster represented its importance in the PPI network, among which Module 1 contained 35 genes (Score = 31.294) (Figure 4C), Module 2 contained 25 genes (Score = 9.25) (Figure 4D), Module 3 contained 14 genes (Score = 5.692) (Figure 4E), Module 4 contained 6 genes (Score = 5.6) (Figure 4F), and Module 5 contained 9 genes (Score = 5.25) (Figure 4G). The three key genes were all in the Module 1 gene cluster with the highest MCODE score. These PPI network analysis results showed that the key genes CXCL8, PTGS2 and IL10 played a key regulatory role in the PPI network, suggesting that they may play an important role in the occurrence and development of gout.

### 3.5 Expression analysis and ROC analysis of key genes

**FIGURE 5:**
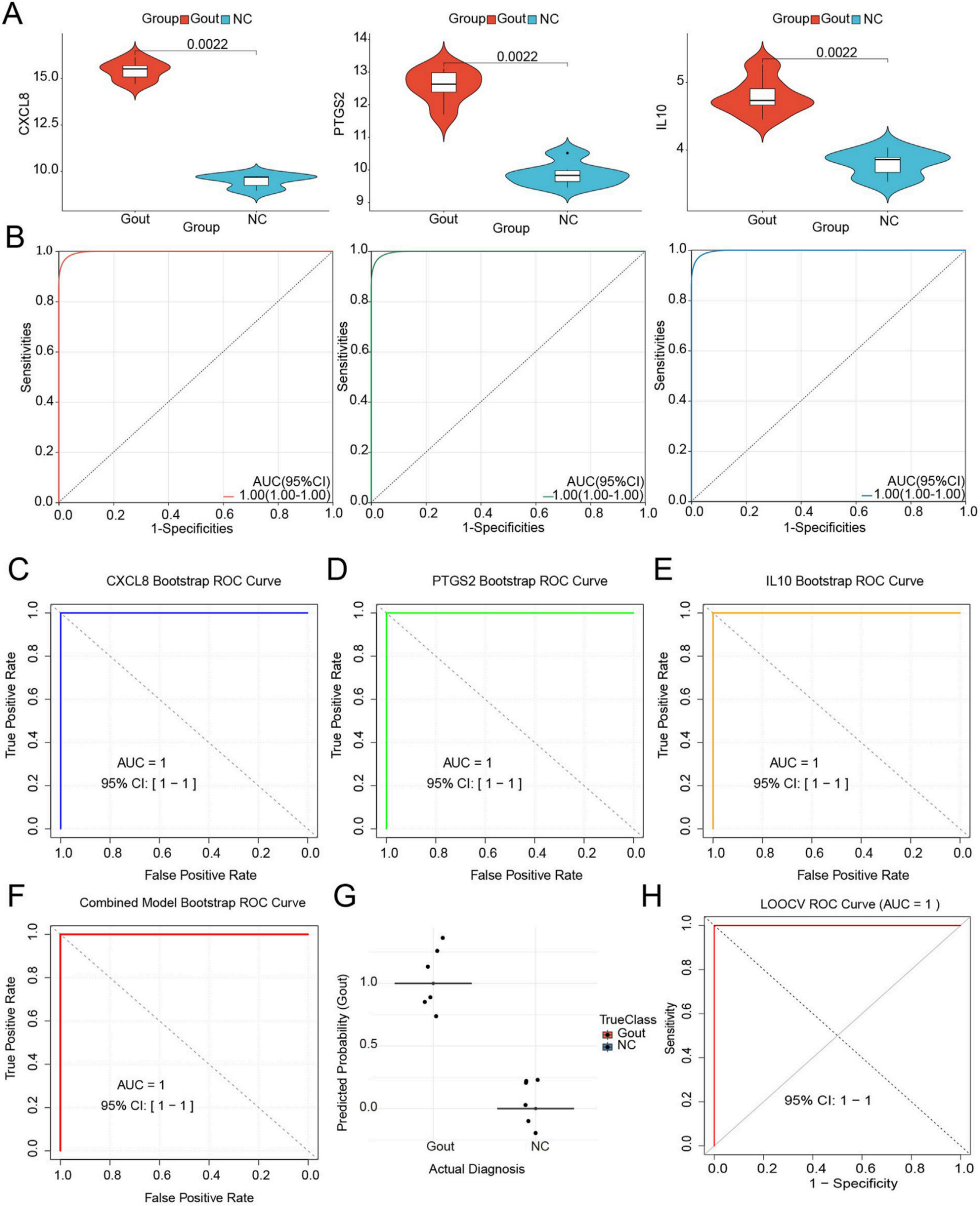
Expression analysis and ROC analysis of key genes. **(A)** Expression analysis of key genes CXCL8, PTGS2 and IL10. **(B)** Receiver operating characteristic (ROC) curves evaluating the diagnostic performance of key genes in distinguishing gout patients from healthy controls. **(C)** Bootstrap validation of key genes CXCL8. **(D)** Bootstrap validation of key genes PTGS2. **(E)** Bootstrap validation of key genes IL10. **(F)** Bootstrap validation of all key genes. **(G)** Distribution of prediction scores by actual diagnosis. Each dot represents an individual sample. Horizontal lines show group means. **(H)** ROC curve from LOOCV analysis.

Based on the transcriptome dataset, the expression patterns and diagnostic performance of key genes (CXCL8, PTGS2, and IL10) were further analyzed. Wilcoxon rank-sum test results demonstrated that compared with the normal control group, all three diagnostic markers in the gout group exhibited significant upregulation (*P* < 0.05) (Figure 5A), suggesting their potential role in promoting gout pathogenesis. ROC analysis revealed that the AUC values for distinguishing gout samples from normal controls reached 1.0 for all genes (Figure 5B), indicating excellent diagnostic capability. Bootstrap validation was performed using 1000 iterations for the three-gene model (CXCL8, PTGS2, and IL10). The analysis yielded a median AUC of 1.0 (IQR: 1–1) (Figures 5C–E), demonstrating consistent perfect discriminative ability across all resampling iterations. The coefficient of variation for all gene coefficient estimates remained below 0.05, indicating stable weighting of the diagnostic markers. The 95% confidence interval calculated using the percentile method was (Gu et al., 2023Gu et al., 2023) (Figure 5F). LOOCV analysis confirmed the robust discriminatory capacity of the three-gene panel, achieving an AUC of 1.00 (95% CI: 1.00–1.00) with complete separation between gout and control groups. All gout samples were correctly classified with prediction probabilities exceeding 0.99, while all control samples demonstrated probabilities below 0.01 (Figure 5G). Coefficient stability analysis across LOOCV iterations showed minimal variation (interquartile range <0.05 for all genes), confirming consistent feature importance rankings. These findings corroborated the bootstrap validation results (AUC = 1.00), establishing model reliability within the current sample constraints, although external validation with larger cohorts remains necessary for clinical translation (Figure 5H).

### 3.6 Construction of nomogram risk prediction model

**FIGURE 6:**
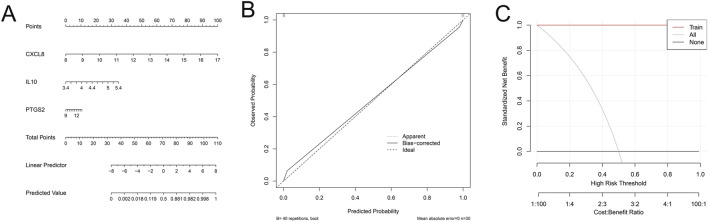
Nomogram risk prediction model. **(A)** Nomogram for predicting the risk of gout based on the expression levels of key genes (CXCL8, PTGS2, and IL10). **(B)** Calibration curve assessing the consistency between the predicted probabilities and the observed outcomes. **(C)** Decision curve analysis (DCA) evaluating the clinical utility of the nomogram.

A nomogram risk prediction model was constructed based on these 3 key genes to assess the risk of gout. The results showed that low expression of key genes CXCL8, PTGS2 and IL10 were risk factors for gout (Figure 6A). Then, to evaluate the accuracy and clinical application value of the nomogram risk prediction model, we plotted the calibration curve and decision curve. The calibration curve showed good consistency between the predicted probability and the actual occurrence probability, indicating that the model had high prediction accuracy (Figure 6B). In addition, DCA was used to evaluate the clinical benefit of the nomogram. The results showed that within a relatively wide range of threshold probabilities, the net benefit of using the nomogram for prediction was better than the two extreme cases of all patients or all non-patients, confirming the potential application value of the nomogram risk prediction model in clinical decision-making (Figure 6C).

### 3.7 Immune infiltration

**FIGURE 7:**
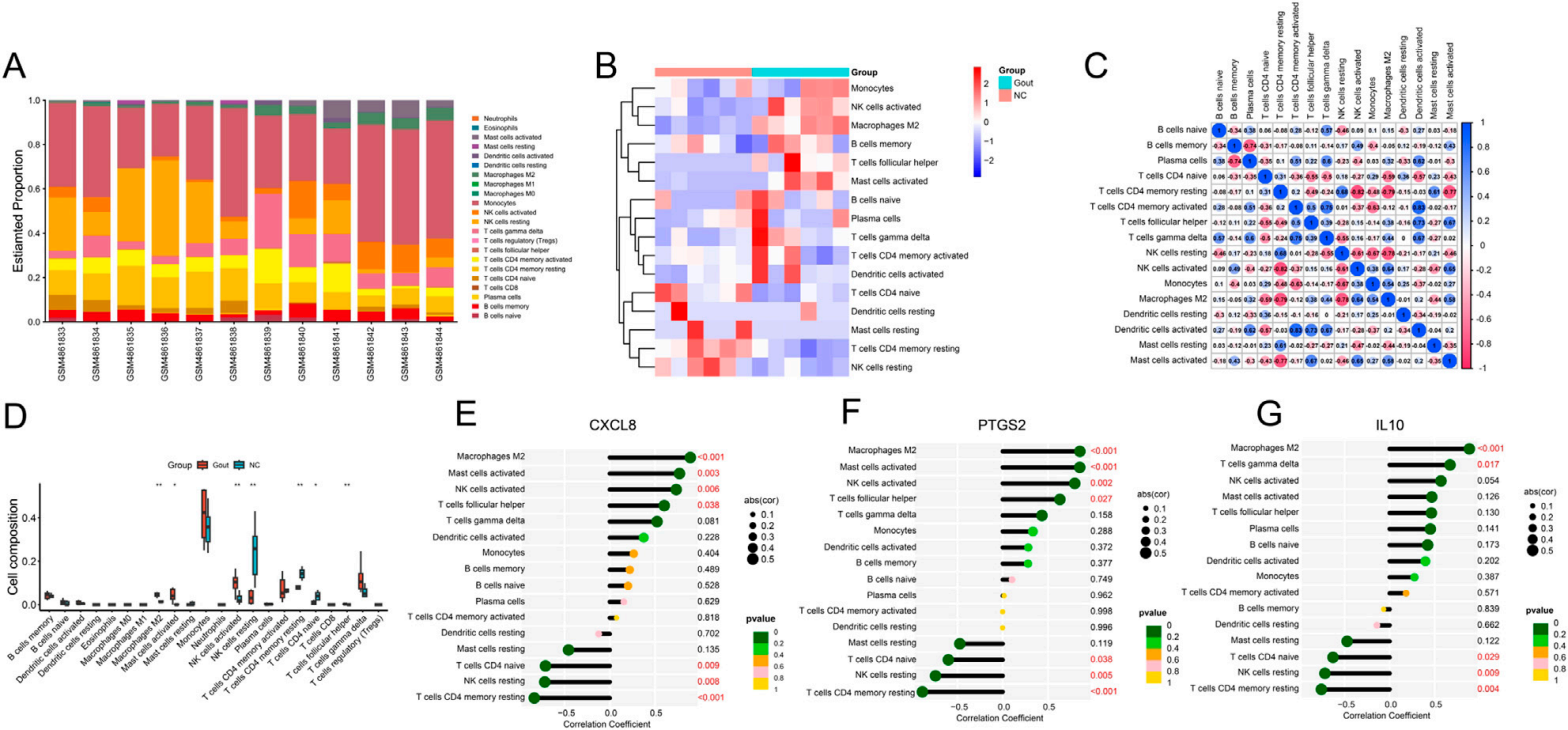
Immune cell infiltration analysis. **(A)** Stacked bar plot showing the proportions of different immune cell subsets in each sample. **(B)** Heatmap displaying the infiltration levels of immune cell subsets across samples. **(C)** Correlation heatmap illustrating the relationships among different immune cell subsets. **(D)** Differential analysis of immune cell infiltration ratio between Gout and NC samples. **(E)** Lollipop plot of correlation between key genes CXCL8 **(E)**, PTGS2 **(F)** and PTGS2 **(G)** and immune cells.

Enrichment analysis showed that immunity is crucial to the development of this disease, so we used the CIBERSORT method to study the infiltration of immune cells in NC and Gout group samples. A total of 16 types of immune cells were identified in NC and Gout group samples. The bar chart and heat map showed the types and numbers of immune cell infiltration in each sample (Figures 7A,B). Then, we assessed the correlation between these immune cell populations. The results showed that dendritic cells activated had the strongest positive correlation with activated CD4 memory T cells (r = 0.83), and resting CD4 memory T cells had the strongest negative correlation with activated NK cells (r = −0.82) (Figure 7C). Then, we compared the infiltration differences of each immune cell subset between the Gout and NC groups. The results showed that four immune cell subsets, M2 macrophages, activated mast cells, activated NK cells, and T cells gamma delta, were significantly upregulated in Gout samples (*P* < 0.05); resting NK cells, resting CD4 memory T cells, and naive CD4 T cells were significantly downregulated in Gout samples (P < 0.05) (Figure 7D). In addition, we further studied the correlation between the expression of the 3 key genes and the infiltration of each immune cell subset. The results showed that these 3 key genes had significant correlations with the infiltration of immune cell subsets such as M2 macrophages, activated mast cells, resting NK cells, resting CD4 memory T cells, and T cells gamma delta. Among them, CXCL8 and PTGS2 had significant positive correlations with M2 macrophages and activated mast cells, IL10 had significant positive correlations with M2 macrophages and T cells gamma delta, and CXCL8, PTGS2 and IL10 had significant negative correlations with resting CD4 memory T cells, resting NK cells and naive CD4 T cells (*P* < 0.05) (Figures 7E–G), suggesting that key genes may play an important role in the occurrence and development of gout by regulating these immune cells.

### 3.8 Drug prediction and molecular docking

**FIGURE 8:**
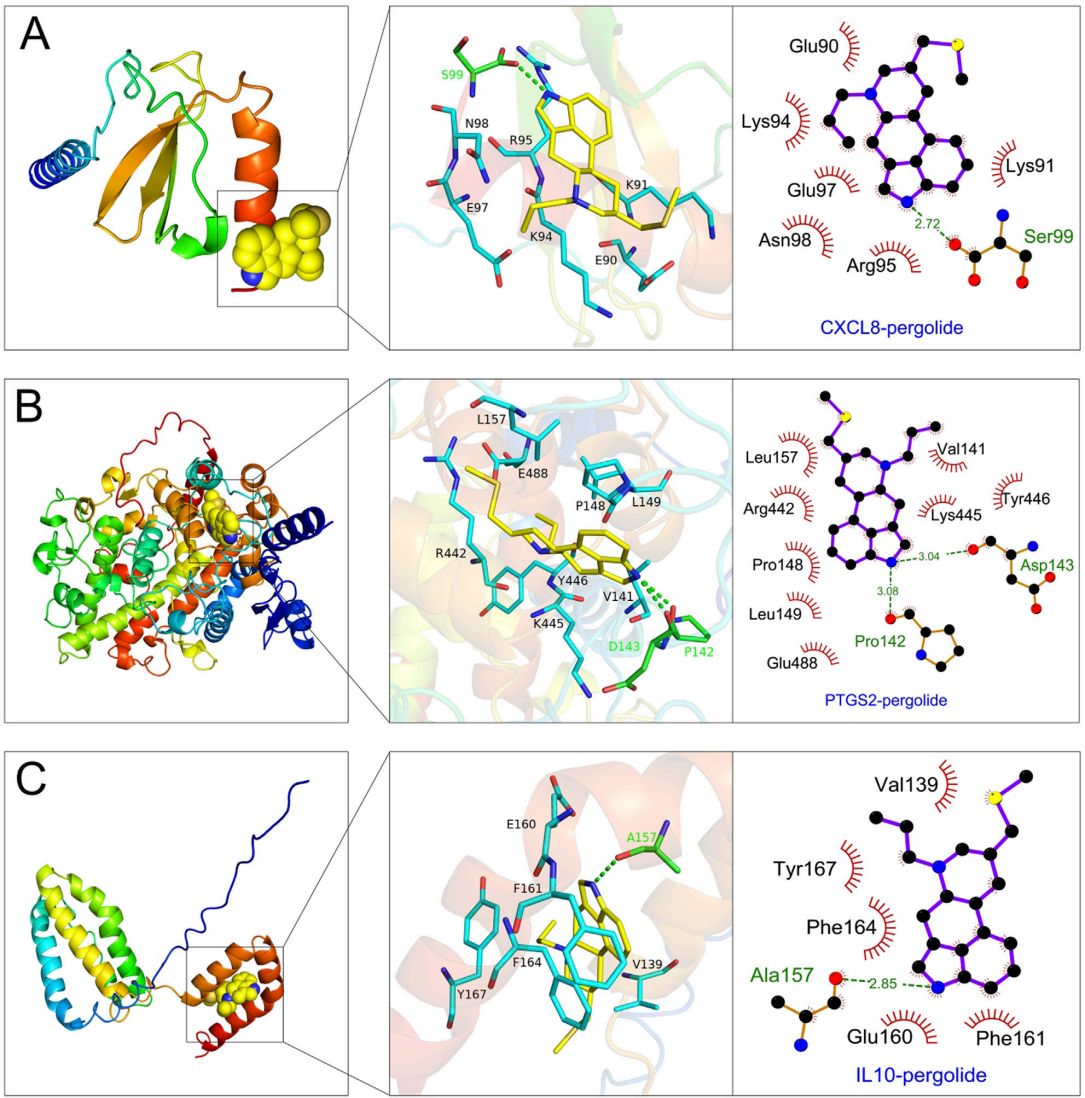
Molecular docking analysis of small molecule drugs and key targets. **(A–C)** Visualization of the binding interactions between pergolide and key targets: CXCL8 **(A)**, PTGS2 **(B)**, and IL10 **(C)**.

The 329 common genes (including 126 upregulated genes and 203 downregulated genes) were uploaded to the CMap platform to screen potential drugs that could improve gout. The results showed that we obtained 5 most potential small molecule drugs (with the largest negative connectivity score and the most significant), namely, enoxacin, selumetinib, d-mannitol, pergolide and roxithromycin. Molecular docking experiments were used to analyze the interaction strength and mode of action between small molecule drugs and key targets. The results showed that all small molecule drugs had good interaction relationships with key targets, and their binding energies were all less than −4 kcal/mol (Table 1). Among these small molecule drugs, pergolide had the best binding with the key targets CXCL8, PTGS2 and IL10, with a binding energy of −7.263 kcal/mol between pergolide and CXCL8, −8.41 kcal/mol between pergolide and PTGS2, and -7.62 kcal/mol between pergolide and IL10. Figure 8 shows the binding patterns between pergolide and the key targets CXCL8, PTGS2 and IL10, with the ligand and receptor connected by multiple hydrogen bonds and hydrophobic bonds, showing good binding. These results suggest that pergolide may act as a targeted drug for key targets to improve gout.

**TABLE 1:** Binding free energies between pergolide and key targets (-kcal/mol).

Ligand Receptor	CXCL8	PTGS2	IL10
pergolide	−6. 393	−7.433	−6.735
pergolide	−6.324	−7.846	−6.391
pergolide	−4.686	−6.319	−4.635
pergolide	−7.263	−8.41	−7.62
pergolide	−6.387	−7.995	−7.503

### 3.9 CeRNA network construction and TF prediction

**FIGURE 9:**
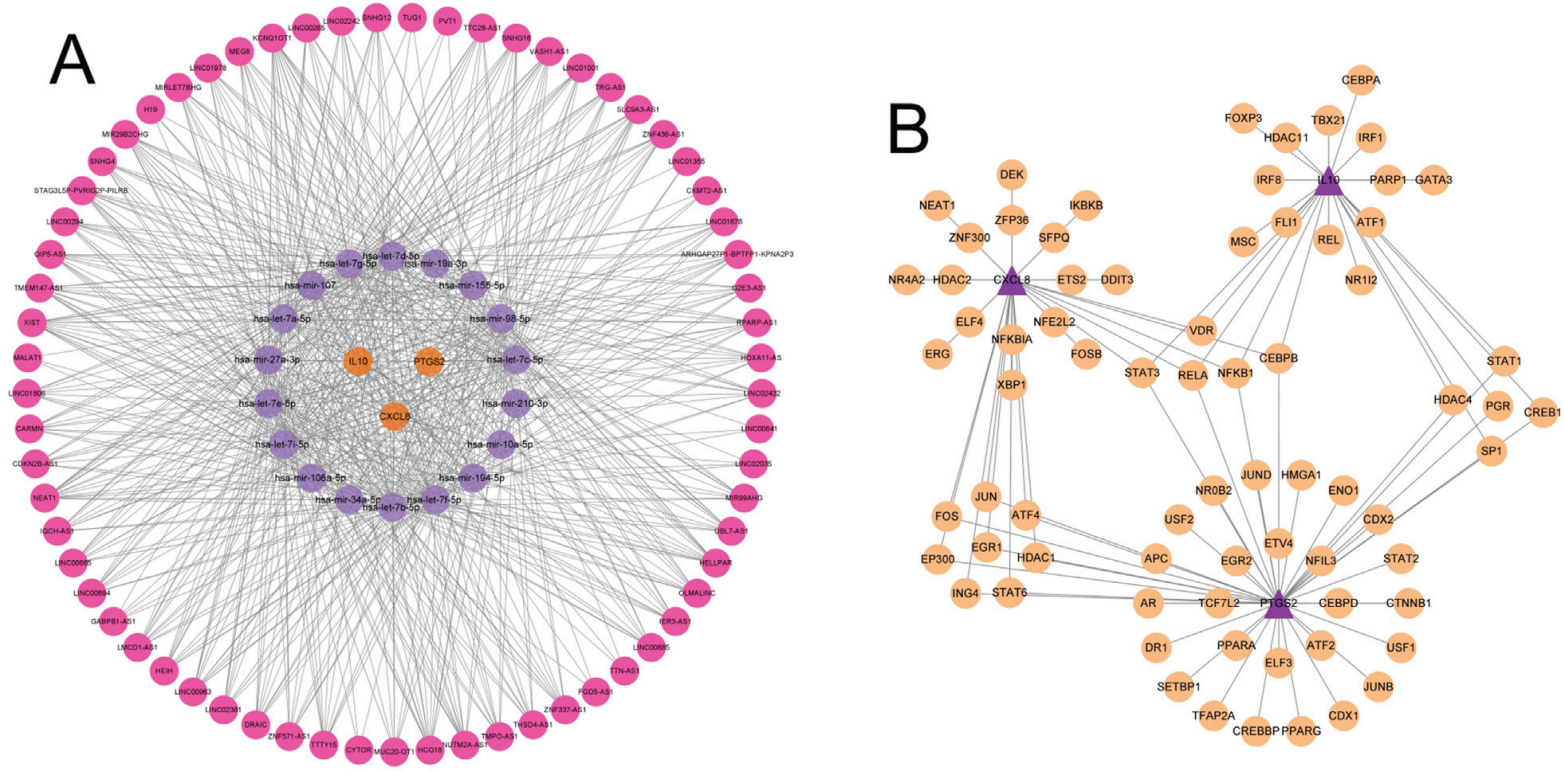
Construction of the ceRNA network and prediction of transcription factors. **(A)** The ceRNA regulatory network. Red nodes represent lncRNAs, purple nodes represent miRNAs, and orange nodes represent mRNAs. Edges indicate the regulatory relationships between the nodes. **(B)** Network of predicted transcription factors and key genes. Light orange nodes represent transcription factors, and purple nodes represent key genes. Edges indicate the regulatory relationships between transcription factors and key genes.

To explore the molecular regulatory mechanism of key genes, the miRNe database was used to predict miRNAs and lncRNAs related to key genes, and a lncRNA-miRNA-mRNA regulatory network (ceRNA network) was drawn. The ceRNA network included a total of 86 nodes (3 key mRNAs, 18 miRNAs and 65 lncRNAs) and 652 edges (interaction relationships). The topological parameters of the ceRNA network were calculated. KCNQ1OT1 was the lncRNA with the largest Degree value in the ceRNA network topology; hsa-mir-98-5p was the miRNA with the largest Degree value in the ceRNA network; IL10, CXCL8 and PTGS2 were mRNAs with the same Degree value in the ceRNA network, and there were mutual regulatory effects between these lncRNAs, miRNAs and mRNAs (Figure 9A), suggesting that these ceRNA networks may be key ceRNA network mechanisms in gout.

Next, we performed transcription factor enrichment analysis on the 3 key genes. The results showed that a total of 76 potential transcription factor binding sites were significantly enriched in the promoter region of key genes in the JASPAR database, among which CEBPB, STAT3, RELA and NFKB1 were common transcription factors of the 3 key genes, suggesting that they may play an important role in the regulation of gout key genes (Figure 9B).

### 3.10 Single-cell data processing and cell clustering

**FIGURE 10:**
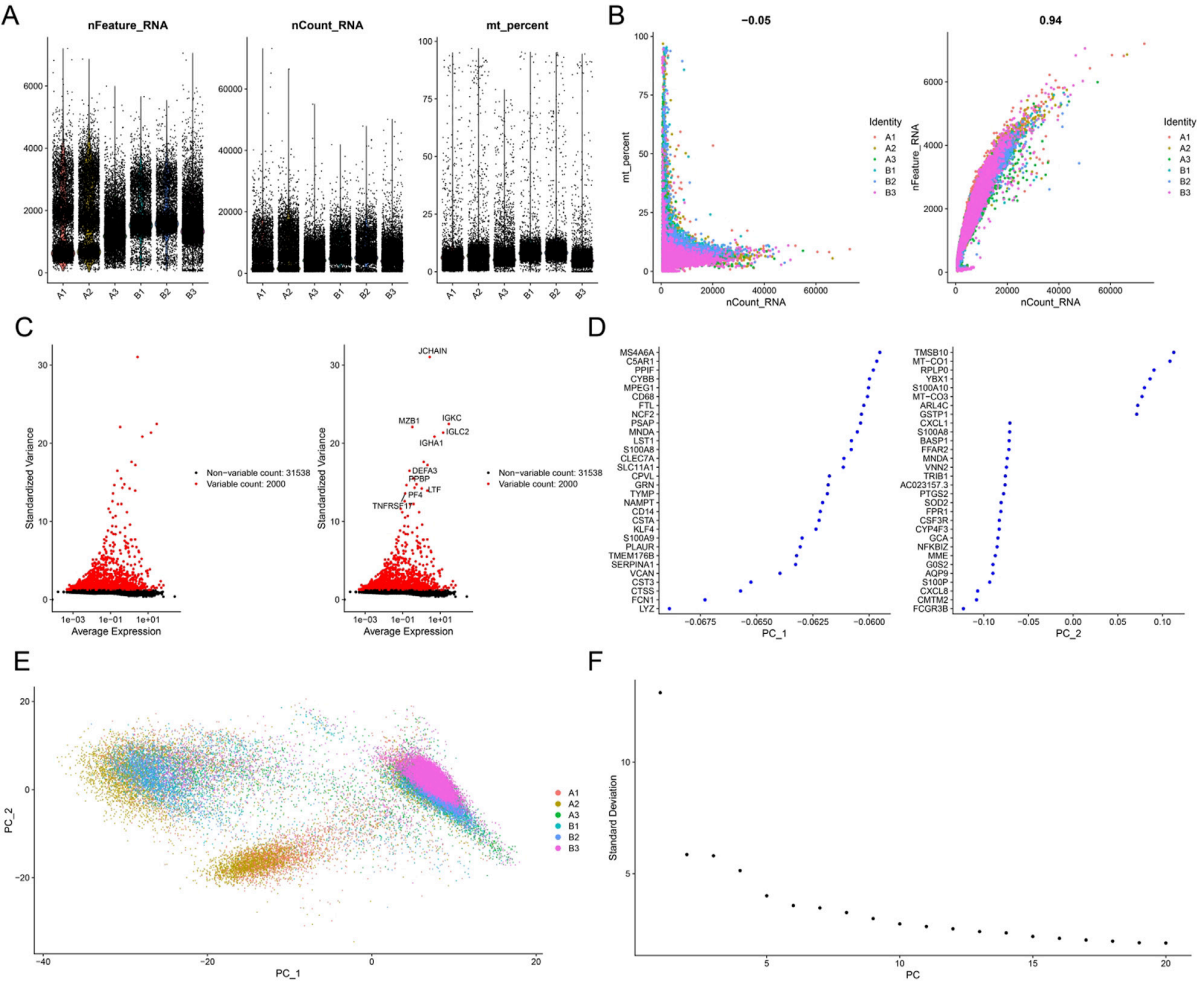
Preprocessing and quality control of single-cell RNA sequencing data. **(A)** Scatter plot of the number of detected genes and the percentage of mitochondrial genes per cell. **(B)** Principal component analysis plot of single-cell gene expression profiles of the normal group and Gout group. **(C)** Feature selection of highly variable genes. Red dots represent highly variable genes, and black dots represent non-variable genes. **(D)** TOP30 genes with cell differentiation. **(E)** PCA plot of single-cell gene expression data. **(F)** Elbow plot showing the standard deviations of the first 50 principal components (PCs).

**FIGURE 11:**
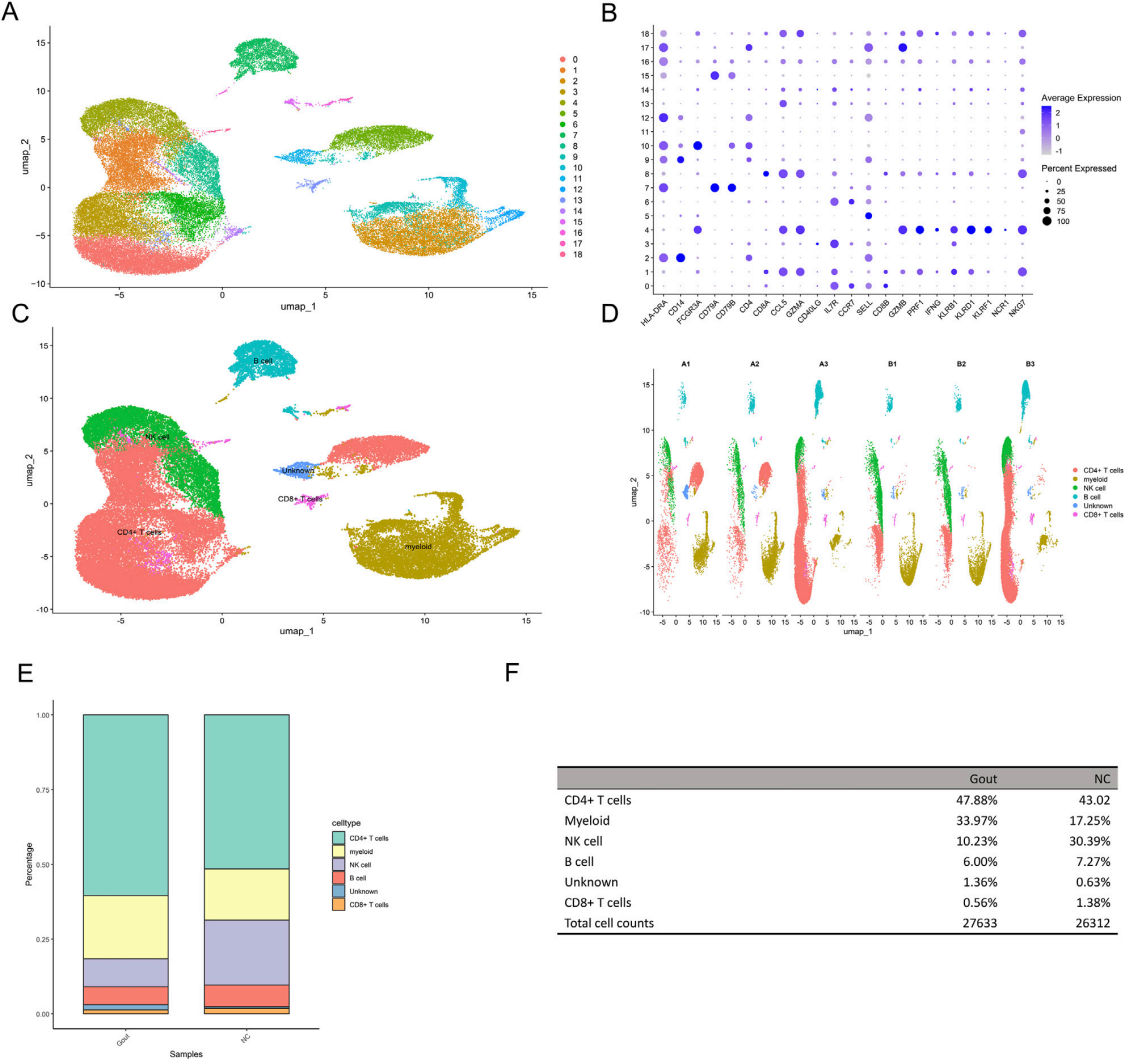
Dimensionality reduction and clustering analysis of single-cell data. **(A)** UMAP plot showing the clustering results of single cells. Each dot represents a single cell, and colors indicate different clusters. **(B)** Dot plot displaying the expression of characteristic genes in each cluster. **(C)** uMAP dimensionality reduction cell annotation. **(D)** uMAP dimensionality reduction clustering result plot of each sample grouping. **(E)** Stacked bar plot showing the proportions of different cell types in the Gout group and the NC group. **(F)** Table summarizing the proportions of different cell types in the Gout group and the NC group.

**FIGURE 12:**
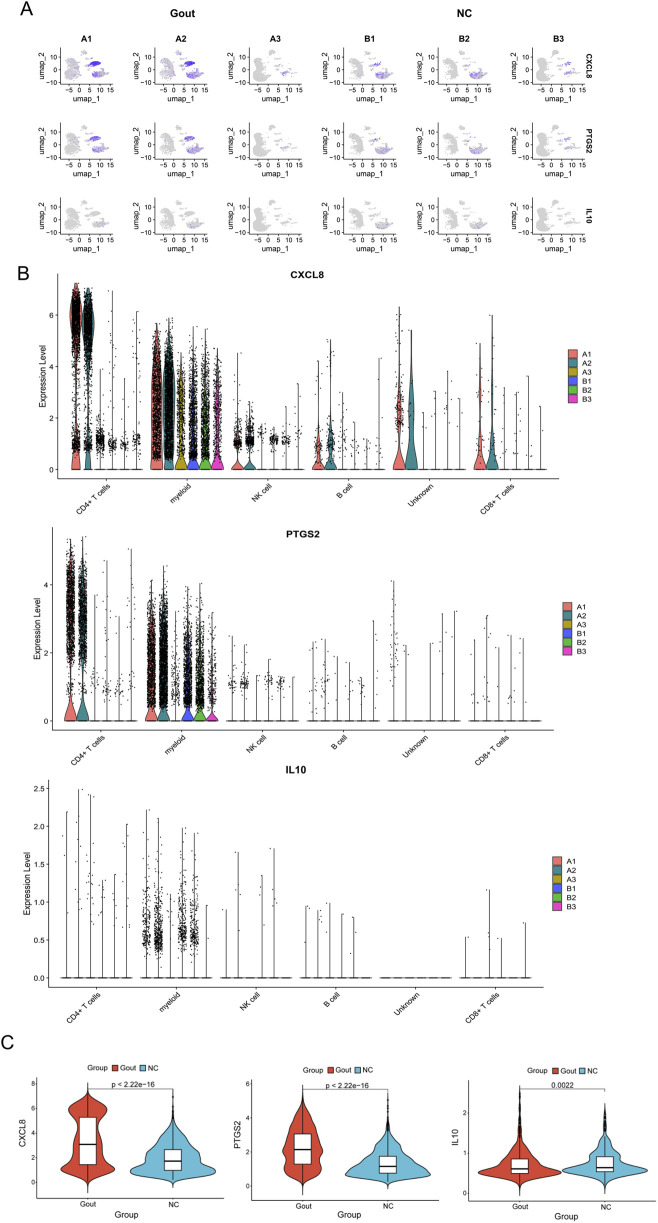
Distribution and validation of key genes in single-cell data. **(A)** UMAP plots showing the expression of key genes (CXCL8, PTGS2, and IL10) in different cell subpopulations in the Gout group and the NC group. Each dot represents a single cell, and the color intensity represents the normalized expression level. **(B)** Violin plots comparing the expression levels of key genes (CXCL8, PTGS2, and IL10) across different cell types. **(C)** Violin plots comparing the expression levels of key genes (CXCL8, PTGS2, and IL10) between the Gout group and the NC group.

The GSE211783 single-cell dataset containing three normal and three gout samples was obtained from the GEO database. The “Seurat” package was used to perform quality control, filtering out cells that did not meet the criteria (nFeature_RNA >200 and nFeature_RNA <7000 and percent.mt < 20), and the resulting core cells were normalized for subsequent analysis (Figures 10A,B). Next, variance analysis was performed to identify highly variable genes, and PCA was conducted on the merged samples (Figures 10C–E). The first 18 PCs were selected for downstream analysis (Figure 10F). Using the UMAP algorithm, the core cells were clustered into 19 independent cell subsets (Figure 11A). Cell clusters were manually annotated based on marker genes identified through literature review, and the expression of key marker genes for each cell type was visualized using bubble plots (Figure 11B). Based on manual annotation, the cells were mainly divided into B cells, myeloid cells, and T/NK cell subgroups. Furthermore, the T/NK cells were subdivided into CD4^+^ T cells, CD8^+^ T cells, and NK cells (Figures 11C,D). We then calculated the proportion of each cell type in the Gout and NC samples. The results showed significant differences in the numbers of CD4^+^ T cells, CD8^+^ T cells, NK cells, and myeloid cells between the Gout and NC groups. Specifically, the proportion of myeloid cells in the Gout group was significantly higher than that in the NC group, while the proportions of CD4^+^ T cells, CD8^+^ T cells, and NK cells were significantly lower, suggesting that the imbalance in these cell populations may be a risk factor for gout (Figures 11E,F). In addition, we analyzed the expression patterns of key genes in the Gout and NC groups and their distribution among the annotated cell types. The results showed that the key genes CXCL8, PTGS2, and IL10 were significantly upregulated in the Gout group compared with the NC group, consistent with the differential expression trends observed in the training dataset (Figures 12A,C). Among them, CXCL8 and PTGS2 were highly expressed and mainly distributed in CD4^+^ T cells, CD8^+^ T cells, NK cells, and myeloid cells, whereas IL10 had lower expression levels and was primarily distributed in myeloid cells (Figure 12B).

### 3.11 Key gene expression validation and function

**FIGURE 13:**
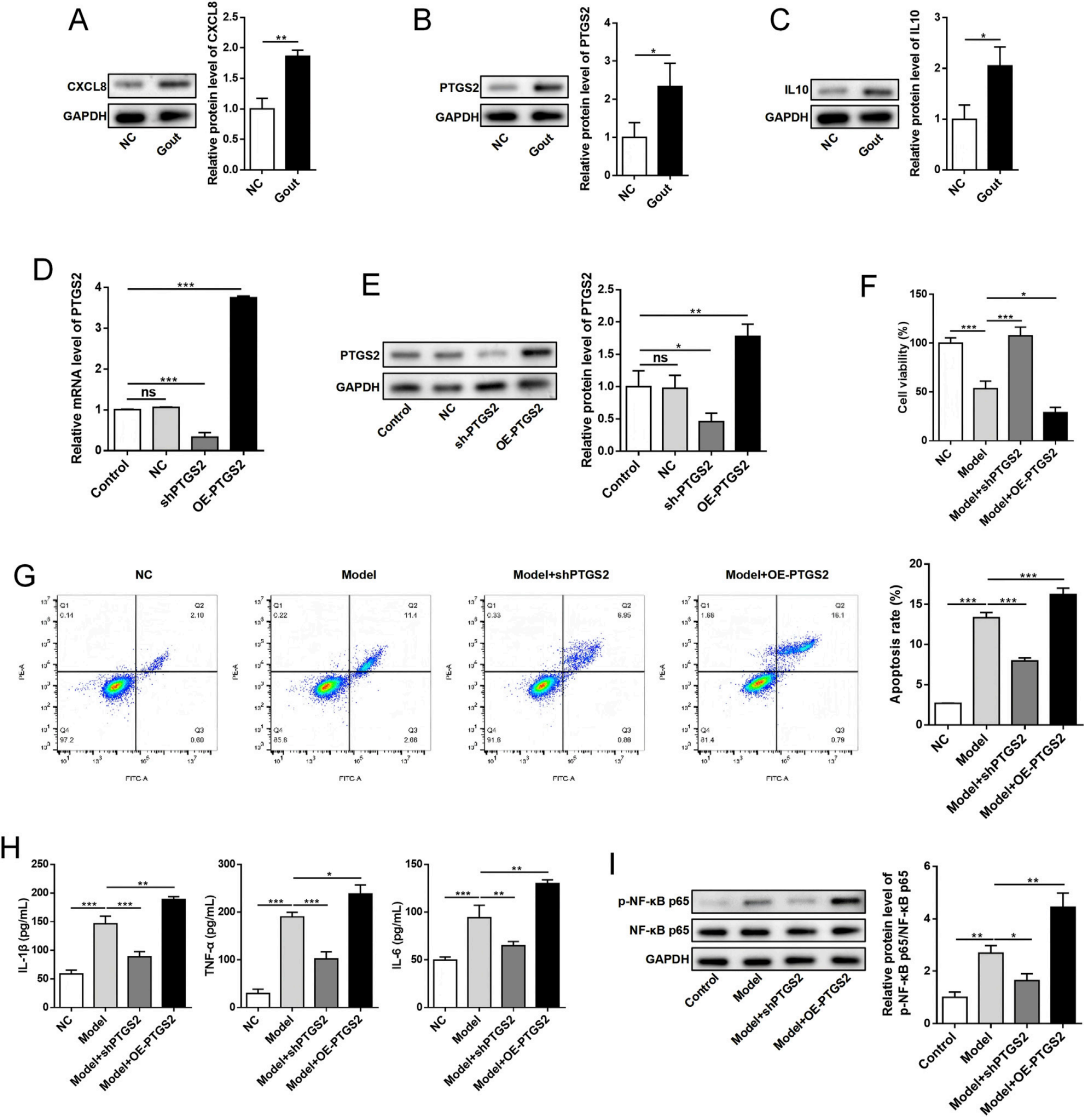
Experimental validation of key gene expression and functional characterization of PTGS2. **(A–C)** Western blot analysis showing protein expression levels of key genes CXCL8, PTGS2, and IL10 in normal control (NC) and gout model groups with quantitative analysis. **(D,E)** PTGS2 transfection efficiency validation by qRT-PCR and Western blot analysis in different treatment groups: Control (non-transfection), NC (negative control), sh-PTGS2 (knockdown), and OE-PTGS2 (overexpression). **(F)** Cell viability measured by CCK-8 assay showing the effects of PTGS2 modulation. **(G)** Flow cytometry analysis of cell apoptosis using Annexin V-FITC/PI staining with representative scatter plots and quantitative analysis. **(H)** ELISA analysis of inflammatory cytokine concentrations (IL-1β, TNF-α, and IL-6) in cell culture supernatants from different treatment groups. **(I)** NF-κB signaling pathway analysis showing Western blot of p-NF-κB p65 and total NF-κB p65 with quantitative analysis of phosphorylation ratio. GAPDH served as internal control. Data are presented as mean ± standard deviation from triplicate experiments. *P < 0.05, **P < 0.01, ***P < 0.001, ns: not significant.

To validate the bioinformatics findings, we conducted experimental analyses using human synovial cells treated with MSU crystals. Western blot analysis confirmed that protein expression levels of all three key genes (CXCL8, PTGS2, and IL10) were significantly upregulated in the MSU-treated group compared to controls (*P* < 0.05), validating our transcriptomic results (Figures 13A–C). Functional characterization of PTGS2 through gain- and loss-of-function studies demonstrated successful transfection efficiency, with PTGS2 mRNA and protein expression significantly decreased in the sh-PTGS2 group (*P* < 0.05) and increased in the OE-PTGS2 group (*P* < 0.05) compared to controls (Figures 13D,E). Cell viability assays and flow cytometric analysis revealed that PTGS2 knockdown enhanced cell survival and reduced apoptosis rates (*P* < 0.05), while overexpression promoted cell death (Figures 13F,G). Furthermore, ELISA analysis demonstrated that PTGS2 overexpression significantly promoted the secretion of pro-inflammatory cytokines IL-1β, TNF-α, and IL-6 (*P* < 0.05), whereas PTGS2 knockdown reduced their production (*P* < 0.05), while Western blot analysis showed that PTGS2 overexpression increased phosphorylation of NF-κB p65 (*P* < 0.05) and PTGS2 knockdown decreased NF-κB activation, indicating the critical role of PTGS2 in regulating this inflammatory signaling pathway (Figures 13H,I). These experimental results collectively demonstrate that PTGS2 functions as a key regulator in gout-associated inflammation by modulating cell survival, promoting pro-inflammatory cytokine production, and activating the NF-κB signaling pathway, providing mechanistic insights into its role in gout pathogenesis and supporting its potential as a therapeutic target.

## 4 Discussion

Gout is a common metabolic disease with a complex pathogenesis that has not yet been fully elucidated. This study adopted a multi-omics joint analysis strategy to systematically mine key genes and their regulatory mechanisms related to the development of gout from the perspective of transcriptomics and single-cell transcriptomics, providing new insights for in-depth understanding of the molecular mechanisms of gout.

Based on the transcriptome dataset, through the integration of differential expression analysis and WGCNA, this study screened out 329 genes closely related to gout. GO and KEGG functional enrichment analyses showed that these genes were mainly enriched in biological processes and signaling pathways related to cell adhesion, immune response, and inflammatory response, such as positive regulation of cell−cell adhesion, positive regulation of leukocyte activation, CCXCR chemokine receptor binding, and NF−kappa B signaling pathway. Intercellular adhesion molecule-1 (ICAM-1), as an immunoglobulin-like cell adhesion molecule, can directly participate in processes such as cell-cell adhesion and leukocyte migration (van de Stolpe and van der Saag, 1996). Studies have found that monosodium urate crystals (MSU) can induce the expression of ICAM-1 in renal mesangial cells, increasing the cell adhesion between renal mesangial cells and human monocytic cells (THP-1), leading to the aggravation of gouty inflammation (Luo et al., 2020). Positive regulation of leukocyte activation refers to the enhancement of the activation state of leukocytes through molecules and signaling pathways. Studies have found that the level of SHP decreases in gout mice, negatively regulating the activation of NLRP3 inflammasome, which may affect the migration of leukocytes and inflammatory response during leukocyte activation (Yang et al., 2015). Inhibition of PI3Kγ reduces the activation of caspase-1 and the recruitment of leukocytes at inflammatory sites in acute gouty arthritis (Tavares et al., 2019). Interleukins can promote cell growth, differentiation, and functional activation, playing a certain role in the positive regulation of leukocyte activation (Mizel, 1989). Interleukins can also regulate the inflammatory response and activation of immune cells in gout. For example, IL-8 is associated with the recruitment and activation of neutrophils (Terkeltaub et al., 1998). IL-1β is a key regulatory factor in gout inflammation, and the main process is: MSU activates the NLRP3 inflammasome, stimulating caspase-1 to promote the activation and secretion of IL-1β, inducing an inflammatory response (Klück et al., 2021). CCXCR chemokine receptor binding is the binding process of CXC chemokine receptors and chemokines of this specific type. The CXC chemokine is a complex system, and it has been confirmed that the levels of factors such as CXCL8, CXCL2, and CXCL8 are significantly elevated in gout patients (Wang et al., 2024), among which CXCL5 activates CXCR2 expressed on nociceptive sensory neurons to trigger TRPA1 activation, leading to inflammatory responses and pain in gouty arthritis (Yin et al., 2024); CXCL2 is associated with inducing neutrophil migration (Ryckman et al., 2003). The NF-κB signaling pathway plays a core role in the inflammatory response of gout. MSU crystals activate the NLRP3 inflammasome and the transcription of pro-IL-1β through the TLR/MyD88-NF-κB signaling pathway, and then promote the maturation and release of IL-1β through the activation of caspase-1 (Liu et al., 2024). In addition, MSU can act together with interferon-γ (IFN-γ) to enhance the production of NO in macrophages by activating the NF-KB and ERK1/2 MAPK pathways, thereby promoting the production of inflammatory cytokines (Jaramillo et al., 2004).

Further PPI network analysis jointly identified three key genes, CXCL8, PTGS2, and IL10, which have a high degree of connectivity in the PPI network, suggesting that they may play a core regulatory role in the pathogenesis of gout. ROC analysis showed that these genes can well distinguish gout patients from healthy controls, and are expected to become markers for the diagnosis and prognosis of gout. The risk prediction model constructed based on key genes has good predictive efficacy and clinical application potential. CXCL8, also known as interleukin-8 (IL-8), belongs to the CXC chemokine family, and is produced by phagocytes and mesenchymal cells exposed to inflammatory stimuli (such as interleukin-1 or tumor necrosis factor), which can attract neutrophils and other immune cells to inflammatory sites and activate neutrophils (Baggiolini and Clark-Lewis, 1992). CXCL8 is a broad-spectrum inflammatory marker that is elevated in rheumatoid arthritis (RA) (Proost et al., 2006), reflecting the degree of neutrophil-mediated inflammation. In gout, monosodium urate crystals can induce the production of IL-8, which binds to the CXCR-2 receptor, leading to the recruitment and activation of neutrophils, and the production of inflammatory mediators such as leukotriene B4 (LTB4) and platelet-activating factor (PAF), exacerbating the inflammatory response of gout (Terkeltaub et al., 1998). PTGS2, also known as prostaglandin-endoperoxide synthase 2, is also called COX-2. COX-2 is a key enzyme in the initial step of PGE2 synthesis, while mPGEs-1 is the final enzyme that converts PGH2 to PGE2. PGE2 is an important inflammatory mediator that plays a role in various physiological and pathological processes (Maione et al., 2020). Studies have shown that the inflammatory response of gout is driven by neutrophils and inflammatory monocytes, which self-sustain local inflammatory responses and maintain inflammatory cycles by activating the preferential coupling of the inducible enzymes COX-2/mPGES-1 and the regulation of PPARγ, while IL-17A neutralizing antibodies can affect this process (Saviano et al., 2022). PTGS2 has shown high diagnostic value in gout, but its expression pattern in other inflammatory arthropathies has not been reported, suggesting that the gene has potential for targeted treatment of gout. It is worth noting that IL-10, as a key anti-inflammatory cytokine, showed an upregulated expression trend in this study, which seems to contradict the previous view that IL-10 mainly exerts anti-inflammatory effects. In fact, IL-10 has a dual regulatory role in the inflammatory response. On the one hand, IL-10 can inhibit the production of pro-inflammatory factors such as IL-12 and IL-18 by monocytes and dendritic cells, and induce T cell differentiation into Th2 type, thereby inhibiting Th1 type inflammatory response (Neumann et al., 2019; Saraiva et al., 2020). On the other hand, IL-10 can also promote the proliferation, differentiation, and antibody production of B cells by up-regulating the expression of co-stimulatory molecules such as CD80 and CD86 on the surface of B cells, aggravating the humoral immune response (Inaba et al., 2023; Cerqueira et al., 2019). In addition, IL-10 can also promote the recruitment of monocytes to the lesion site by up-regulating the expression of monocyte chemoattractant protein 1 (MCP-1), aggravating local inflammatory responses (Nikaein et al., 2023). IL10 shows complex regulatory patterns in different inflammatory arthropathies. Its overexpression has been fully demonstrated in RA (Hernández-Bello et al., 2017), but the specific mechanism of action in gout needs further study. Experimental validation further supported our bioinformatics results. Protein expression of CXCL8, PTGS2, and IL10 was significantly upregulated in MSU-treated synovial cells, consistent with transcriptomic findings. Functional assays showed that PTGS2 knockdown improved cell viability and reduced apoptosis, while overexpression promoted pro-inflammatory cytokine production (IL-1β, TNF-α, IL-6) and activated NF-κB signaling. These results highlight PTGS2’s key role in mediating inflammatory responses in gout and suggest it may serve as a promising therapeutic target.

Immune cell infiltration analysis found that M2 macrophage, activated mast cells, activated NK cells, and γδT cells were significantly upregulated in gout tissues, suggesting that they may be involved in the pathological process of gout. The expression of key genes was significantly correlated with the degree of infiltration of these immune cells, suggesting that there may be important mutual regulation between the two. M2 macrophage, as one of the polarization phenotypes of macrophages, plays an important role in the remission period of gout. M2 macrophage produces anti-inflammatory cytokines, inhibits the progression of inflammation, and promotes tissue repair (Di Vito Nolfi et al., 2022). M2 macrophage can remove MSU crystals in a “non-inflammatory phagocytosis” manner, and can also secrete cytokines with anti-inflammatory effects such as IL-4 and TGFβ, participating in the remission of inflammation, tissue remodeling, and angiogenesis (Sica and Mantovani, 2012). After mast cell activation, a large number of mediators can be secreted, including stored products (such as histamine and trypsin) as well as many cytokines, including IL-1β (Stack and Johnson, 1994). Activated mast cells are involved in the early stages of crystal-induced inflammation, releasing inflammatory mediators such as histamine in response to C3a, C5a, and IL-1. At the same time, vasodilation, increased permeability, and pain are also mediated by kinins, complement cleavage peptides and other vasoactive substances such as prostaglandins (Choi et al., 2005). One study found tryptase and histamine in the synovial fluid of acute gout attacks. These mast cell-related mediators are usually stored in mast cell granules and released when mast cells are activated, indicating that activated mast cells occur during acute gout attacks. The study also showed that there were high levels of mast cell-derived IL-1β in the synovial fluid of gout patients, further confirming that mast cells are involved in the inflammatory response in gout patients (Reber et al., 2014). Mast cells also contribute to the initiation of gouty inflammation. Ablation of mast cells in a mouse model of MSU crystal-induced peritonitis significantly reduced neutrophil recruitment (Getting et al., 1997). Natural killer (NK) cells are innate lymphocytes that participate in defending against pathogens and cancer cells in the body (Campbell and Hasegawa, 2013). NK cells are divided into two functionally different subsets according to CD56 expression levels: CD56dim subset with effective cytotoxicity and CD56bright subset with poor cytotoxicity that secretes a large number of cytokines (Lanier et al., 1986; Jacobs et al., 2001; Cooper et al., 2001). NK cells in the joints of gout patients show an increased CD56 bright group, which can produce a large number of pro-inflammatory and anti-inflammatory cytokines. At the same time, NK cells can also participate in the spontaneous regression stage of gout. NK cells can express anti-inflammatory cytokines, and have also been shown to limit pro-inflammatory monocyte activity by killing highly active cells (Nedvetzki et al., 2007; van Dommelen et al., 2006). γδT cells are a subset of T cells with unique T cell receptors (TCRs), mainly including two major subsets, namely, Vγ9/Vδ2 and Vδ1, and different subsets have different immune functions (Morita et al., 1991). One study showed that γδT cells are one of the main sources of IL-17 in the serum of patients with acute gouty arthritis (AGA) (Liu et al., 2018), while IL-17 is an effective pro-inflammatory cytokine, and its elevated levels are associated with the progression of autoimmune diseases and cancer (Iwakura et al., 2011), showing that γδT cells may be involved in the pathogenesis of inflammatory responses in AGA patients. A recent study showed that γδ2T cells may also migrate to the synovial fluid of AGA patients by interacting with chemokine receptors (CXCR3) and secreting pro-inflammatory cytokines (IL-17) to participate in the mechanism of gout (Di et al., 2024). In addition, in order to reveal the post-transcriptional regulatory mechanism of CXCL8, PTGS2, and IL10, this study constructed a gout-related ceRNA regulatory network based on the miRNet database, and identified a series of lncRNAs (such as KCNQ1OT1) and miRNAs (such as miR-98-5p) that may play an important regulatory role in the pathogenesis of gout. LncRNAs affect gene expression through multiple mechanisms, including competitive binding with miRNAs, affecting chromatin structure, and transcription factor activity. A recent literature showed that KCNQ1OT1 showed upregulation and downregulation effects on the inflammatory response in patients with osteoarthritis by interacting with miR-126-5p and miR-211-5p, respectively (Taheri et al., 2024). KCNQ1OT1 binds to miR-98-5p by adsorbing it, thereby competing with miR-98-5p for the binding sites of its target genes, relieving the inhibitory effect of miR-98-5p on its target genes, thus demonstrating the expression of gout-related target genes. Transcription factor enrichment analysis revealed the upstream transcriptional regulatory network of CXCL8, PTGS2, and IL10. The results showed that key transcription factors such as CEBPB, STAT3, RELA, and NFKB1 may be involved in the pathogenesis of gout by directly regulating the transcriptional levels of these genes. Among them, CEBPB (CCAAT/enhancer-binding protein beta) is an important inflammation-related transcription factor that can respond to various inflammatory stimuli (such as TNF-α and IL-1β) and activate the expression of downstream inflammatory genes (Ren et al., 2023). STAT3 is another key transcription factor involved in inflammation and immune regulation, which can regulate the balance of pro-inflammatory cytokines and anti-inflammatory cytokines through multiple signaling pathways (such as the JAK/STAT pathway) (Hu et al., 2023). RELA and NFKB1 are the core components of the classic NF-κB signaling pathway, directly involved in the regulation of various pro-inflammatory genes (such as CXCL8 and PTGS2), driving gout-related inflammatory responses (Ngo et al., 2020; Xu et al., 2022).

To identify potential therapeutic compounds for gout, we performed small molecule drug prediction analysis using the CMap database. The analysis revealed five promising candidate drugs: enoxacin, selumetinib, d-mannitol, pergolide, and roxithromycin. Among these, pergolide exhibited the highest binding affinity with key gene-encoded proteins, suggesting potential therapeutic effects against gout through targeting CXCL8, PTGS2, and IL10. While pergolide is primarily used for Parkinson’s disease treatment via dopamine D2 receptor stimulation, no previous studies have investigated its role in gout (Clarke and Speller, 2000). Our molecular docking results provide theoretical foundation for pergolide as a potential gout therapeutic.

Single-cell analysis revealed distinct cellular compositions between gout and control groups. The gout group showed significantly increased myeloid cell proportions and decreased T/NK cell proportions, suggesting that myeloid cell infiltration and T/NK cell exhaustion may drive gout pathogenesis. CXCL8 and PTGS2 were predominantly expressed in T/NK cells and myeloid cells, while IL10 was mainly expressed in myeloid cells, with all key genes showing elevated expression in the gout group. These results are consistent with previous findings that myeloid cells, especially monocytes/macrophages and neutrophils, play key roles in gout via NLRP3 inflammasome activation and IL-1β secretion (Liu et al., 2022; He et al., 2019). The reduction in T/NK cells may result from excessive activation and exhaustion driven by pro-inflammatory mediators like CXCL8 (Park et al., 2024; Carvalho et al., 2022).

Although this study systematically elucidated the molecular mechanisms underlying gout through a multi-omics integration analysis, some limitations remain. The main limitation is the small sample size, which may cause individual differences to overly influence the results. Future research should increase the sample size to improve data representativeness and statistical power. Additionally, while pergolide was preliminarily identified as a potential therapeutic drug through bioinformatics and molecular docking, large-scale clinical studies are needed to confirm its efficacy and clinical value in gout treatment.

In summary, this study adopted a strategy of integrating multi-omics data analysis to systematically explain the key roles and regulatory mechanisms of CXCL8, PTGS2, and IL10 in the pathogenesis of gout, which not only deepens the understanding of the pathogenesis of gout, but also provides new ideas and potential targets for developing molecular diagnostic markers and new treatment strategies for gout. In the future, larger-sample, multi-center translational medical research needs to be carried out to ultimately realize the translation of research results into clinical applications.

## Data Availability

Publicly available datasets were analyzed in this study. This data can be found here: https://www.ncbi.nlm.nih.gov/geoprofiles/.
